# Dr. Edward Eckerson

**Published:** 1915-02

**Authors:** 


**Dr. Edward Eckerson,** Buffalo, 1877, died at his home in Denver, December 14, of cerebral haemorrhage, aged 61. He had practiced in Denver for a quarter of a century.

